# An ERp57-mediated disulphide exchange promotes the interaction between *Burkholderia cenocepacia* and epithelial respiratory cells

**DOI:** 10.1038/srep21140

**Published:** 2016-02-16

**Authors:** Francesca Pacello, Melania D’Orazio, Andrea Battistoni

**Affiliations:** 1 Department of Biology, University of Rome Tor Vergata, Roma, Italy

## Abstract

Previous studies have demonstrated that extracellular glutathione reduces the ability of the Cystic Fibrosis pathogen *Burkholderia cenocepacia* to infect primary or immortalized epithelial respiratory cells. We report here that the adhesion and invasion ability of *B. cenocepacia* is limited also by thiol-oxidizing and disulphide-reducing agents and by protein disulfide isomerase (PDI) inhibitors. PDI inhibitors also reduce the proinflammatory response elicited by cells in response to *Burkholderia*. These findings indicate that a membrane-associated PDI catalyzes thiol/disulphide exchange reactions which favor bacterial infection. The combined use of selective PDI inhibitors, RNA silencing and specific antibodies identified ERp57 as a major PDI involved in the interaction between *B. cenocepacia* and epithelial cells. This study contributes to the elucidation of the *Burkholderia* pathogenic mechanisms by showing that this microorganism exploits a membrane-associated host protein to infect epithelial cells and identifies ERp57 as a putative pharmacological target for the treatment of *Burkholderia* lung infections.

A well known feature of Cystic Fibrosis (CF) is the marked decrease of reduced glutathione (GSH) concentration in the airway surface liquid (ASL) of patients^1^,^2^. This defect is the consequence of a reduced export of GSH through the lung epithelium and of an abnormal consumption of this antioxidant due to sustained chronic inflammation. In fact, some *in vitro* studies have suggested that the chloride efflux CFTR channel, which belongs to the MRP/ABC family of proteins that includes several GSH transporters, could be the direct mediator of GSH export^3^,^4^. The importance of a functional CFTR channel for GSH export is confirmed by the observation that CFTR knockout mice show comparable alterations in GSH extracellular content^5^ and fail to adapt GSH levels in response to cigarette smoke^6^. At the same time, other studies have revealed that low concentrations of GSH in the airways of young CF patients are associated to high levels of glutathionylated proteins and of glutathione sulfonamide, a specific byproduct of the reaction of GSH with the hypochlorous acid released *in vivo* by the abundant neutrophiles recruited in the CF lung^7^. Moreover, GSH^7^ and protein^8^ oxidation increases in CF children during pulmonary infections.

The role of extracellular GSH in the lung has been the object of limited investigations, but it is likely that it contributes to the control of lung inflammation by protecting the lung tissue by the damage caused by the reactive oxygen species spontaneously generated in this highly oxidizing environment or actively produced by neutrophils^1^,^9^. In addition, extracellular GSH could modulate mucus viscosity and regulate the redox state of membrane proteins containing labile disulphides^10^. There is also some evidence suggesting that extracellular GSH has a role in the response to bacterial lung infections. For example, GSH can reduce the toxic effects of pyocyanin^11^,^12^,^13^, a redox-active exotoxin released in large quantities by *Pseudomonas aeruginosa* during lung infections^14^, which significantly contributes to the pathophysiological alterations typical of the CF lung^15^. The concentration of GSH in the ASL significantly increases in wild type mice following *P. aeruginosa* infection, whereas this response is not observed in CFTR mutant mice^16^. Moreover, there is evidence that mycoplasma infections inhibit GSH adaptive response to oxidative stress^17^. We have recently demonstrated that GSH can drastically reduce the ability of the CF pathogen *Burkholderia cenocepacia* to adhere and invade epithelial respiratory cells, including CFTR deficient primary cells isolated from the lung of a CF patient undergoing to organ transplant^18^. The reduced ability of bacteria to interact with host cells is correlated with a drastic reduction of the inflammatory response and to an increase of free thiol groups on the proteins located on the external cell membrane^18^. This observation is suggestive of a GSH-mediated change in the redox status of membrane proteins involved in *B. cenocepacia* recognition. Among the membrane-associated proteins which could be affected by changes in the GSH levels outside the cells there are members of the Protein Disulphide Isomerase (PDI) family. PDIs are typically localized in the endoplasmic reticulum, where they contribute to the maturation of newly synthesized proteins by catalyzing the formation and reshuffling of disulphide bonds^19^. However, several studies have revealed that some PDIs may be found also in other subcellular districts (cytoplasm, nucleus, cell membrane) where they may functionally contribute to a variety of cellular activities^20^,^21^. Membrane-associated PDIs have been implicated in the attachment and entry of several viruses^22^,^23^,^24^,^25^,^26^, of bacteria of the *Chlamydia* genus^27^,^28^ of the protozoan *Leishmania chagasi*^29^, as well as in the uptake of peptides and proteins, including diphtheria and cholera toxins^30^,^31^. These studies have suggested that PDI may mediate the transient formation of disulfide bonds between a membrane receptor and a protein located on the surface of the infectious agent, thus facilitating its subsequent internalization. As high levels of extracellular GSH could affect PDI activity and/or the structure of receptor proteins, we have investigated the possibility that *Burkholderia* adhesion and infection are promoted by host PDIs.

## Results

### Thiol-modifying reagents reduce the invasive ability of *B. cenocepacia* LMG 16656

**Figure 1:**
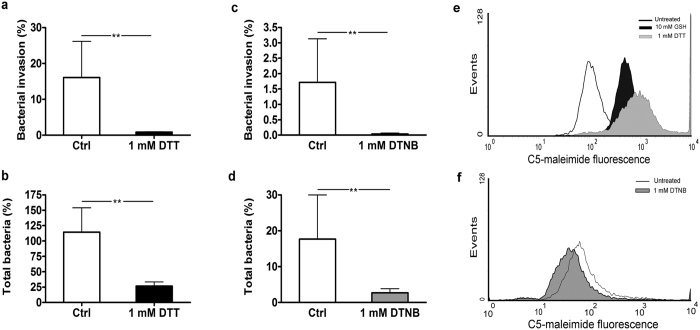
Both DTT and DTNB reduce the ability of *B. cenocepacia* LMG 16656 to infect epithelial cells. (**a)** Invasion of 9HTEo- cells by *B. cenocepacia* LMG 16656 after 3 hours of infection in presence or absence of 1 mM DTT. (**b)** Effect of 1 mM DTT on the total number of bacteria (adherent + intracellular) interacting with 9HTEo- epithelial cells after 3 hours of infection. (**c)** Invasion of 9HTEo- cells by *B. cenocepacia* LMG 16656 after 1 hour of infection in presence or absence of 1 mM DTNB. (**d)** Effect of 1 mM DTNB on the total number of bacteria (adherent + intracellular bacteria) interacting with 9HTEo- epithelial cells after 1 hour of infection. The data reported in (panels **a–d**) represent the mean ± SD of 3 independent experiments. Asterisks denote statistically significant results (**p < 0.001). (**e,f)** Cytofluorimetric analysis of surface thiols after treatment with DTT and GSH (**e**) and DTNB (**f**). Cells were incubated for 3 hours with 1 mM DTT or with 1 mM GSH (**e**) or for 1 hour with 1 mM DTNB (**f**) and then treated with 10 μM Alexa fluor C_5_-maleimide to label surface free thiols and then analyzed by FACScalibur system, as described in Materials and Methods. The histograms from a typical experiment are shown.

To test the hypothesis that extracellular GSH interferes with *B. cenocepacia* ability to infect epithelial respiratory cells by modifying cysteine residues of cell surface proteins^18^, we have carried out invasion assays in presence of the reducing agent dithiotreithol (DTT) or of the membrane-impermeant thiol oxidant 5,5′ dithio-bis (2-nitrobenzoic)acid (DTNB) which forms mixed disulfides with –SH groups^32^. Figure 1a shows that when 9HTEo- cells were infected for 3 hour with *B. cenocepacia* LMG 16656 in presence of 1 mM DTT, there was a more than 90% decrease in the number of intracellular bacteria with respect to control cells. Similarly, also the number of total *B. cenocepacia* LMG 16656 associated to 9HTEo- cells (including either adherent or intracellular bacteria) was markedly modified by the incubation with 1 mM DTT (Fig. 1b), indicating that DTT affects either adhesion or invasion of *B. cenocepacia.* At the same time, the oxidation of free thiols resulting from incubation of 9HTEo- cells with 1 mM DTNB induces a strong decrease of *B. cenocepacia* LMG16656 invasion (approximately 98%) and adhesion (85%), as shown in Fig. 1c,d, respectively. Incubation of 9HTEo- cells with 1 mM DTT enhanced the number of cell surface thiols to levels which are even higher to those observed after a treatment with 10 mM GSH^18^, whereas incubation with DTNB caused a reduction of surface-associate thiols (Fig. 1e,f).

**Figure 2:**
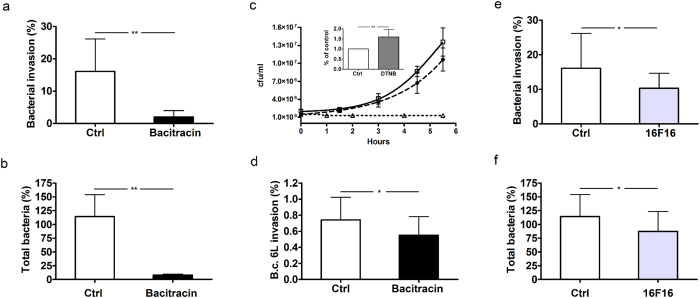
PDI inhibitors decrease the ability of *B. cenocepacia* LMG 16656 to infect 9HTEo- cells. **(a,b)** Effect of 0.3 mM bacitracin on. *B. cenocepacia* invasion (panel **a)** and on the total number of bacteria (adherent + intracellular) interacting with 9HTEo- cells (panel **b)**. (**c)** Effect of bacitracin (open square, solid line) or DTNB (open triangle, dotted line) or the absence of treatment (filled circle, dashed line) on *B. cenocepacia* growth in MEM supplemented with 2 mM glutamine and 2% FBS. The inset shows the effect of bacterial pretreatment with 1 mM DTNB on *B.c.* LMG 16656 invasiveness into 9HTEo- cells. **(d)** Inhibition of *B. cenocepacia* 6L invasion in 9HTEo- cells in presence of 0.3 mM bacitracin. (**e,f**) Effect of 10 μM 16F16 on *B. cenocepacia* invasion (panel **e**) and on the total number of bacteria interacting with epithelial cells (panel **f)**. Each panel reports the mean ± SD of, respectively, 4 (panels **a**,**b**), 5 (panel **c**) or 3 (panels **d–f**) independent experiments. Asterisks denote statistically significant results: *p < 0.05; **p < 0.001.

When infection experiments were carried out using bacteria pretreated with DTNB, but omitting the addition of this chemical agent during the incubation of bacteria with cells, we observed a significant increase in bacterial invasion ability, despite DTNB having a bacteriostatic effect (Fig. 2c). This observation suggests that free thiols located on the bacterial surface hinder *B. cenocepacia* interaction with epithelial cells.

These experiments confirm that cysteine residues of proteins associated to the membranes of epithelial cells and, possibly, on the bacterial surface, play a role in the interaction and/or the entry of *Burkholderia* into host cells and suggest that infectivity of this bacterium is dependent on protein targets prone to cysteine modification either by reducing or oxidizing agents.

### PDI inhibitors are able to influence *B. cenocepacia* ability to invade epithelial cells

Putative protein targets susceptible to both reducing and oxidizing agents include enzymes of the PDI family. To evaluate if PDIs are involved in the interaction of *B. cenocepacia* with 9HTEo- cells, we have used well known inhibitors of enzymes of this class. We have initially tested the effect of bacitracin which is widely used as inhibitor of PDI activity^30^. As shown in Fig. 2a,b, bacitracin significantly inhibits both adhesion and invasion of *B. cenocepacia* LMG 16656 in 9HTEo- cells. The number of intracellular and total bacteria recovered from 9HTEo- cells treated with bacitracin is approximately 90% lower than that of bacteria found in control cells. Interestingly, the extent of bacitracin-mediated inihibition of *B. cenocepacia* invasivity is comparable to that obtained with 10 mM extracellular GSH^18^ or 1 mM DTT.

It must be noted that, although bacitracin has antibacterial activity against Gram-positive bacteria^33^, it has no effects on *Burkholderia* growth (Fig. 2c). Moreover, we have observed a significant increase in the number of extracellular bacteria in the supernatants of cells treated with bacitracin (data not shown), thereby confirming that this drug affects the interaction between bacteria and epithelial respiratory cells.

The effect of bacitracin was also tested on the *B. cenocepacia* 6L clinical isolate, observing that also the ability of this strain to penetrate into 9HTEo- cells is reduced by bacitracin to an extent comparable to that obtained with extracellular GSH^18^ (Fig. 2d). This observation supports the hypothesis that PDI has a role in mediating the ability of different *B. cenocepacia* strains to invade epithelial cells.

To confirm the involvement of enzymes of the PDI family in *B. cenocepacia* interaction with epithelial cells, we have carried out infection experiments in the presence of 16F16, a compound that inhibits the PDI isoforms P4HB (also known as PDIA1) and ERp57 (also known as PDIA3)^34^.

The results reported in Fig. 2e,f show that when the infection was carried out in presence of 10 μM 16F16 there was a significant reduction in the number of intracellular and adherent bacteria with respect to control cells. Higher concentrations of 16F16 proved to be toxic for 9HTEo- cells and could not be tested. As in the case of bacitracin, we did not observe adverse effects of this compound as well as of all the other PDI inhibitors used in this work, except DTNB (Fig. 2c), on *B. cenocepacia* growth (data not shown).

**Figure 3:**
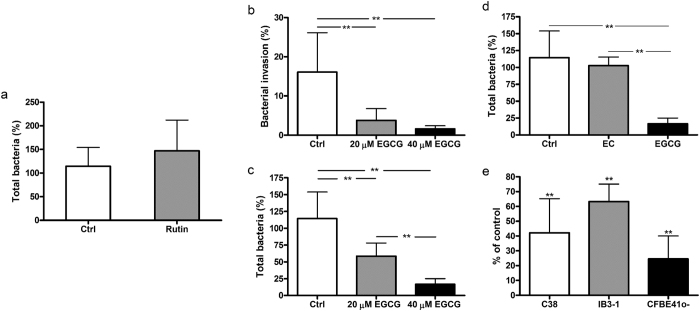
*B. cenocepacia* invasion is specifically inhibited by PDI inhibitors targeting ERp57. **(a)** Effect of 30 μM rutin on *B. cenocepacia* ability to adhere and invade 9HTEo- cells. **(b,c)**. Effect of 20 and 40 μM EGCG on the invasion (panel **b**) or on the total number of bacteria (panel **c**) interacting with 9HTEo-cells. (**d)** Differential effects of epicathechin (EC) and epigallocatechin gallate (EGCG) on *B. cenocepacia* ability to adhere and invade 9HTEo- cells. **(e)** EGCG impairs *B. cenocepacia* invasivity in different epithelial cell lines. The reported data are the mean ± SD of, respectively, 3 (panels **a**,**b**,**d**,**e**) or 8 (panel **c**) independent experiments. Asterisks denote statistically significant results: **p < 0.001.

A specific inhibitor of P4HB is quercetin-3-rutinoside, also known as rutin^35^. Infection experiments carried out in presence or in absence of 30 μM rutin failed to show an effect of this compound on the invasion or adhesion of *B. cenocepacia* to 9HTEo- cells (Fig. 3a). This result suggests that P4HB is not a major PDI isoform mediating the ability of this pathogen to infect epithelial cells.

To evaluate the possible involvement of ERp57, we tested the effects on *B. cenocepacia* invasivity of EGCG, a green tea flavanol which has been shown to bind to this specific PDI isoform *in vitro* and modify its enzymatic activity and DNA-binding properties^36^. 9HTEo- cells have been pre-incubated with 0, 20 or 40 μM EGCG for 30 minutes and then infected with *B. cenocepacia* LMG 16656 for 3 hours in presence of the same amount of EGCG. Panels **b** and **c** of Fig. 3 show that EGCG reduces *B. cenocepacia* infectivity in a concentration-dependent manner. The number of total and intracellular bacteria isolated from cells treated with 40 μM EGCG decreased by 85% and by more than 90%, respectively, whereas this reduction was less marked when 9HTEo- cells were incubated with 20 μM EGCG. The reduction in bacterial infectivity obtained with 40 μM EGCG is comparable to that obtained with 10 mM GSH. No inhibition of the invasive ability of *Burkholderia* was observed using 40 μM epicatechin (Fig. 3d), which, lacking the galloyl moiety, binds ERp57 with low affinity and does not modify its activity^36^.

The effect of EGCG on *B. cenocepacia* LMG 16656 invasion was also tested on other cell lines, such as CFBE41o-, C38 and IB3-1. In all these cell lines we observed a consistent reduction in bacterial invasion when infections were carried out in presence of 40 μM EGCG (Fig. 3e).

These experiments show that a wide range of PDI inhibitors is able to interfere with the capability of *B. cenocepacia* to adhere and invade epithelial cells, and suggest that ERp57 could play an important role in the infectivity of this pathogen.

### Silencing or inhibition of ERp57 reduces *B. cenocepacia* ability to penetrate in epithelial cells

**Figure 4:**
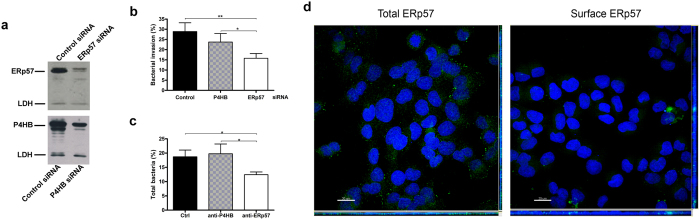
Erp57 is involved in *B. cenocepacia* interaction with respiratory cells. **(a)** Western blot analysis of ERp57 and P4HB in 9HTEo- cells transfected with different siRNAs. Lactate dehydrogenase (LDH) was used as a loading control. (**b)** Invasion assay of *B. cenocepacia* LMG 16656 in 9HTEo- cells transfected with control siRNA, P4HB siRNA and ERp57 siRNA. The reported values are means ± SD obtained by measuring *B. cenocepacia* LMG 16656 invasion ability in a representative experiment, including six replicates. *p < 0.05; **p < 0.001. (**c)** Effect of antibody-mediated PDIs blockade on the ability of *B. cenocepacia* to interact with 9HTEo- cells. Histograms represents the total number of bacteria (adherent + intracellular) recovered from the cell monolayer after 1 hour of infection in presence or in absence of antibodies against ERp57 or P4HB. The reported values are means ± SD from four replicates in a representative experiment. *p < 0.05. **(d)** Identification of a fraction of ERp57 associated to 9HTEo- cell membranes. MIPs (Maximum Intensity Projections) with orthogonal projections XZ (below of each panel) and YZ (right of each panel) from confocal system acquisition (Olympus IX 81 inverted microscope, software FV 1000) of 9HTEo- cells fixed and permeabilized (left panel) and unpermeabilized 9HTEo- cells (right panel). ERp57, detected by immunofluorescence, appears in green, whereas the nuclei, stained with Hoechst, appear in blue. Bars = 20 μm.

To obtain additional evidence of the involvement of ERp57 in the interaction between *Burkholderia* and epithelial cells, we have carried out infection experiments in 9HTEo- cells transfected with short interfering RNA (siRNA) for ERp57, P4HB or with a negative control siRNA. Figure 4 shows that cells exhibiting a decreased level of ERp57 (panel a) are less prone to *B. cenocepacia* invasion (panel b). The number of intracellular bacteria in cells transfected with the ERp57-specific siRNA was 45% lower than in cells transfected with an irrelevant RNA. In contrast, the silencing of P4HB caused only a modest decrease of *Burkholderia* entry into 9HTEo- cells with respect to control siRNA cells, which did not reach statistical significance (Fig. 4b).

To further investigate the role of P4HB and ERp57 in *B. cenocepacia* interaction with 9HTEo-cells, we have carried out infection experiments in presence of 50 μg/ml of a function-blocking anti-P4HB antibody^30^ or of an anti-ERp57 antibody, after pre-treating the cells for 30 minutes with the antibodies. No difference in *Burkholderia* infectivity has been observed between control cells and 9HTEo- cells incubated with the P4HB antibody (Fig. 4c). In contrast, we observed a significant reduction (35%) in the number of bacteria associated to 9HTEo- cells when the infection was carried out in presence of the anti-ERp57 antibody (Fig. 4c).

Together, these experiments show that the PDI isoform ERp57 has a major role in mediating *B. cenocepacia* interaction with epithelial cells, whereas the PDI isoform P4HB has little or no role in this process. Immunofluorescence studies in permeabilized and unpermeabilized cells confirmed that small amounts of ERp57 can be localized on the surface of 9HTEo- cells (Fig. 4d).

### PDI inhibitors reduce the inflammatory response of 9HTEo- cells to infections by *B. cenocepacia* LMG 16656

**Figure 5:**
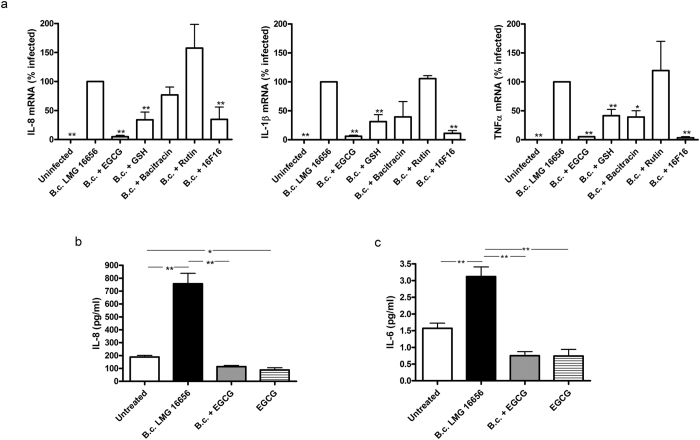
ERp57 inhibitors reduce the expression and release of some pro-inflammatory cytokines in response to *B. cenocepacia* infection. (**a**) The expression of IL-8, TNF-α and IL-1β was analyzed by RT-PCR in 9HTEo- cells infected with *B. cenocepacia* LMG 16656 (10 CFU/cell). Infection was carried out either in cells kept in standard medium or in cells incubated with various chemical agents interfering with PDI activity, including EGCG (40 μM), GSH (10 mM), bacitracin (0,3 mM), rutin (30 μM) and 16F16 (10 μM). Data are the average +/− SD of two independent experiments and the results are shown as relative percentages of expression of each cytokine with respect to its expression in cells infected in the absence of PDI inhibitors. (**b**) Effect of 40 μM EGCG treatment on the IL-8 release from 9HTEo- cells incubated with or without B.c. LMG 16656 for 3 hours. Supernatants of cultures were collected and IL-8 was measured by ELISA. Values represent the mean ± SD of four individual cultures. (**c**) IL-6 release from 9HTEo- cells as described in **b**.

We have previously observed that extracellular GSH reduces the inflammatory response of epithelial cells to *B. cenocepacia* infections^18^. To evaluate the possibility that also inhibitors of PDI may reduce the inflammatory response of 9HTEo-induced by *B. cenocepacia*, we have analyzed by quantitative real-time RT-PCR the expression of IL-8, TNF-α and IL-1β, into tracheo-bronchial cells infected in absence and in presence of 10 mM GSH, 0.3 mM bacitracin, 10 μM 16F16, 30 μM rutin and 40 μM EGCG. With the exception of rutin, all the other PDI inhibitors proved to significantly decrease the *B. cenocepacia-*induced expression of the three Cytokines (Fig. 5). The capacity of EGCG to control the inflammatory response elicited by *B. cenocepacia* was confirmed by its ability to significantly reduce the release of IL-6 and IL-8 (Fig. 5b,c).

## Discussion

CF is characterized by recurrent pulmonary infections caused by opportunistic pathogens that show a marked ability to proliferate in the lungs of patients. *B. cenocepacia* is a typical CF pathogen characterized by an intrinsic multidrug resistance^37^. Although it colonizes a relatively small number of CF patients, it is associated with a negative prognosis because it can promote a condition described as “cepacia syndrome”, which is characterized by rapid pulmonary decline and death^38^.

We have recently observed that extracellular GSH impairs the ability of *B. cenocepacia* to adhere to and penetrate within primary and immortalized epithelial respiratory cells^18^. As GSH is poorly able to permeate membranes, this observation suggested that this reducing agent could modulate the interaction between *Burkholderia* and epithelial cells by affecting the redox state of cysteines and/or disulphides of membrane-associated proteins involved in bacterial recognition. In this work we have provided further support to this hypothesis by showing that other thiol-modifying agents, such as DTT and DTNB, impair *B. cenocepacia* ability to infect cells (Fig. 1). DTNB, just as GSH, is unable to permeate membranes suggesting that these compounds affect *B. cenocepacia* infectivity by altering protein targets localized on the cell surface of epithelial cells. Moreover, either reducing (DTT and GSH) or oxidizing agents (DTNB) have similar effects on bacterial adhesion and invasion. This observation prompted us to investigate the possibility that enzymes of the PDI family, whose activity depends on highly reactive dithiol/disulfide centers, could mediate bacterial binding and internalization of *B. cenocepacia*. Although PDIs are primarily located in the endoplasmic reticulum to favor folding of nascent proteins, there is abundant evidence that a fraction of the enzyme localizes on the cell surface^20^,^21^.

The possibility that a host protein catalyzing the formation and breakage of disulfide bonds mediates *Burkholderia* infection has been initially evaluated in experiments involving the broad spectrum PDI inhibitor bacitracin, which directly interacts with cysteines in the substrate-binding domain of PDI by forming a disulphide bond with an open thiol form of the bacitracin thiazoline ring^39^. Bacitracin was found to inhibit bacterial attachment and entry at levels comparable with GSH^18^ (Fig. 2). However, as the specificity of bacitracin has been questioned by studies showing that it can bind free cysteines also in other proteins^40^, we have tested other structurally unrelated PDI inhibitors. We found that also 16F16 and EGCG reduce the capability of *Burkholderia* to adhere to or enter within 9HTEo- cells. This result is not due to effects of these compounds on the growth or survival of bacteria. It can be noted that an inhibition of *B. cenocepacia* invasivity comparable to that obtained incubating cells with 10 mM GSH^18^ was achieved using 0.3 mM bacitracin or 40 μΜ EGCG (Figs 2 and 3).

The use of multiple inhibitors, besides strengthening the hypothesis that PDI activity mediates *Burkholderia* infection, gives indications on the identity of the PDI isoform facilitating bacterial interaction with respiratory cells. The PDI family, indeed, includes as many as 21 different proteins^41^, many of which have been identified at the cell surface. Whereas bacitracin is a rather unspecific PDI inhibitor, there is evidence that compounds such as 16F16, rutin and EGCG show preferential inhibitory activity towards specific PDI isoforms^34^,^35^,^36^. The observation that *Burkholderia* invasion ability is insensitive to rutin, an inhibitor of the P4HB isoform, but is inhibited by 16F16 and EGCG, which are able to inhibit the ERp57 isoform, prompted us to further investigate the involvement of this specific enzyme in the ability of the pathogen to infect respiratory cells.

The role of ERp57 has been confirmed either by RNAi experiments or by the use of function-blocking antibodies. In fact, we have shown that ERp57-depleted epithelial cells are infected by a lower number of bacteria with respect to control cells (Fig. 4b) and that the incubation with an ERp57 antibody protects 9HTEo- cells from *Burkholderia* adhesion and entry (Fig. 4c). In contrast, the silencing of P4HB leads to a non-significant reduction of bacterial invasion and an anti-P4HB antibody does not modify bacterial interaction with cells. Interestingly, previous studies have shown that antibodies against P4HB impair entry of *Chlamydia* and of different viruses within eukaryotic cells^27^,^42^,^43^. On the contrary, ERp57 is involved in the entry of the rotavirus ECwt^44^, whereas the nairovirus nairobi sheep disease virus/ganjam virus has been recently proved to induce translocation of ERp57 and PDI to the cell membrane during the infection^24^. These studies suggest that distinct PDI isoforms may mediate the recognition of specific infectious agents.

**Figure 6:**
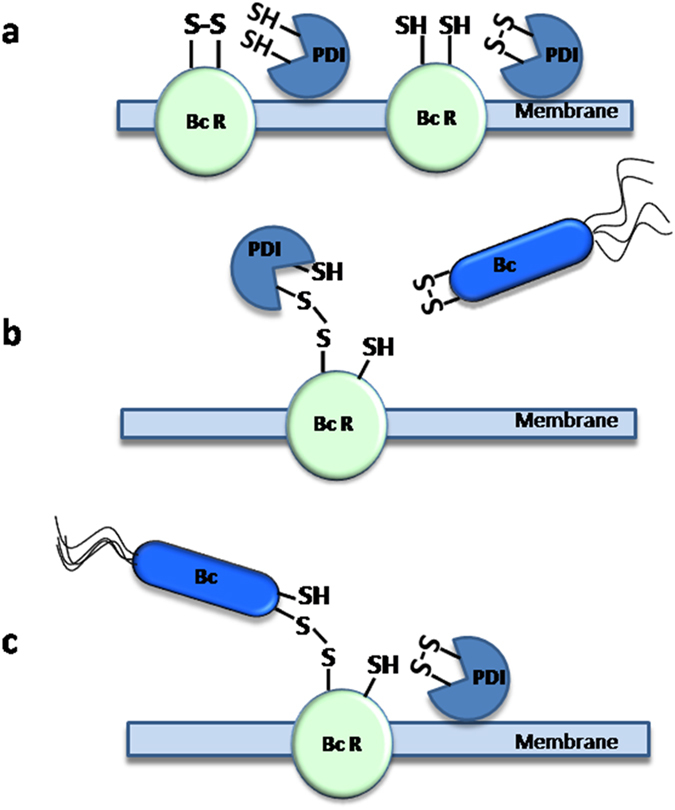
Schematic model for the ERp57-mediated disulphide exchange which favors bacterial attachment to epithelial cells. We suggest that membrane-associated ERp57 (PDI) (panel **a**) catalyzes a disulphide exchange reaction between a surface exposed *Burkholderia cenocepacia* receptor (BcR) and a disulphide bond located on a *B. cenocepacia* (Bc) solvent exposed protein. We hypothesize that the formation of a transient disulphide bond between ERp57 and a BcR located on the cell membrane (panel **b**) promotes *B. cenocepacia* adhesion to the cell surface (panel **c**) and the subsequent internalization of the bacterium.

*Burkholderia* is able to enter within mammalian cells, where it efficiently survives and replicates by its ability to interfere with the antimicrobial responses of the host cell^45^,^46^,^47^. The modality by which *B. cenocepacia* gains access into host cells is not completely understood, but a few membrane proteins involved in the host-*Burkholderia* recognition have been identified. It has been shown that, depending on the specific strain, *Burkholderia* can interact with mucin, cytokeratin 13, tumor necrosis factor receptor 1 (TNFR1), glycolipid and glycosphingolipid receptors present on epithelial cells^45^,^46^. Interestingly, most of the above mentioned putative protein receptors contain disulphide bonds which could be substrates for the activity of ERp57. For example, TNFR1 contains cysteine-rich extracellular domains which are involved in protein conformational stabilization and in control of receptor responsiveness^48^. Additional studies are needed to identify the cell surface receptor(s) involved in the recognition of *B. cenocepacia* and to directly demonstrate that ERp57 has the ability to bind bacteria and/or modulate the oxidoreductive state of thiols of receptors proteins, but our results, collectively, converge in indicating that this pathogen may efficiently penetrate within epithelial cells by exploiting membrane-associated ERp57. Although the physiological role of surface exposed ERp57 in epithelial respiratory cells is not known, it should be noted that previous studies have identified specific functions for the extracellular form of this enzyme in other cell types, including participation to sperm-egg fusion^49^, platelet aggregation^50^, the binding of 1α,25-dihydroxycholecalciferol by gut epithelial cells^51^ and, together with calreticulin, determination of immunogenicity of cell death^52^. We propose that ERp57 represents a “Trojan Horse” which facilitates *Burkholderia* invasion of epithelial cells through a mechanism similar to that schematized in Fig. 6. The suggestion that the target on the bacterial surface is a disulphide bond is supported by the observation that bacterial pretreatments with DTNB stimulate invasion (Fig. 2c, inset), whereas pretreatments with DTT reduce invasion (not shown) and those with GSH have no effects^18^.

In this work we have also shown a correlation between inhibition of ERp57, *Burkholderia* infection and pro-inflammatory response of the infected epithelial cells. ERp57 inhibitors reduce the pro-inflammatory response elicited by 9HTEo- cells in response to *B. cenocepacia* infection. This finding confirms that strategies aimed at reducing *B. cenocepacia* intracellular burden might be of help in controlling the inflammatory response to this pathogen.

This work provides some clues towards possible treatments of *B. cenocepacia* infections in CF patients. Our observations suggest that low levels of GSH in the ASL may contribute to the ability of *B. cenocepacia* to colonize the lung of CF patients by influencing the activity of proteins belonging to the PDI family. Several studies have been carried out to evaluate the efficacy of therapies aimed at elevating the concentration of GSH in the lung of patients, but their effectiveness remains doubtful^53^. It has been suggested that poor efficacy of therapies based on GSH nebulization could be explained by the presence of high levels of the GSH degrading enzyme γ-glutamyl transferarse in the ASL of CF patients^54^. Our studies indicate that inhibitors of PDI activity have an ability to limit *B. cenocepacia* interaction with epithelial cells comparable to GSH, thereby representing a potential therapeutic alternative to favor the control of *B. cenocepacia* infections. It is worth nothing that EGCG, besides showing the ability to inhibit ERp57, has been reported to inhibit biofilm formation by a variety of CF pathogens^55^, to interfere with *P. aeruginosa* quorum sensing^56^; to limit lung colonization by *Stenotrophomonas maltophilia*^57^ and to contribute to the control of viral infections^58^,^59^,^60^,^61^. The combination of these studies and our observations mark EGCG as a potentially promising drug for the treatment of bacterial lung infections in CF and other respiratory diseases.

## Methods

### Reagents

GSH, bacitracin, 16F16, rutin, epigallocatechin gallate (EGCG), epicatechin (EC), dithiotreitol (DTT) and 5,5′ dithio-bis (2-nitrobenzoic)acid (DTNB) were provided from Sigma Aldrich. GSH solutions were prepared as previously described^18^. Bacitracin was dissolved in the infection medium. DTNB was prepared in ethanol, DTT was solubilized in distilled water, whereas 16F16, rutin, EGCG and EC were dissolved in DMSO. Antibodies against P4HB (RL90) and ERp57(sc-23886) were obtained from Thermo scientific and Santa Cruz, respectively. All these compounds were subsequently diluted in the infection medium.

### Bacterial strains and growth conditions

The *B. cenocepacia* strain LMG 16656, corresponding to the sequenced reference strain J2315^62^, was obtained from the Belgian Coordinate Collection of Microorganism (BCCM). The clinical isolate *B. cenocepacia* 6L (*cblA-*; BCESM+)^63^ was chosen for its marked difference in interaction with epithelial cells with respect to *B. cenocepacia* LMG 16656^18^. Bacteria grown on *Pseudomonas* Isolation Agar (PIA) plates (DifcoTM) were inoculated in chemically defined medium (CDM), containing 48 mM glucose, 7.4 mM KCl, 6 mM NaCl, 48 mM (NH_4_)_2_SO_4_, 0.5 mM MgSO_4_×7H_2_O, 60 mM MOPS, 3.8 mM K_2_HPO_4_×3H2O, 0.1% Casamino acid and cultivated without agitation at 37 °C, as previously described^18^.

### Host cell lines and culture conditions

The human tracheobronchial epithelial cell line 9HTEo-64 and the cystic fibrosis human bronchial epithelial cell line CFBE41o- (ΔF508/ΔF508)^65^ were kindly provided by Dr. Dieter Gruenert (University of California at San Francisco). The cystic fibrosis bronchial epithelial cell line IB3−1 (ΔF508/W1282X) and the isogenic CFTR-complemented C38 cell line were obtained from ATCC.

Cells were routinely maintained in MEM (9HTEo-, and CFBE41o-) or D-MEM (IB3-1 and C38) (Euroclone) supplemented with 2 mM glutamine, 100 U/ml penicillin, 0.1 mg/ml streptomycin, and 10% heat-inactivated fetal bovine serum (FBS), in a humidified 5% CO_2_ incubator, at 37 °C. All cell lines were grown in bovine serum albumin-collagen-fibronectin coated flasks and the confluent, adherent monolayers were released from the plastic surface after treatment with trypsin (C38 and IB3-1) or polyvinyl-pirrolidone-trypsin-EDTA solution (9HTEo-, and CFBE41o-), collected by centrifugation at 700 × g and resuspended in fresh medium. The effect of all treatments (bacterial infections, incubation with each chemical compound or with a combination of bacteria and compounds) on cell viability was evaluated by the trypan blue exclusion method, without observing any significant consequence on cell viability.

### Invasion assays

Cell monolayers were prepared by seeding 1.5 × 10^5^ cells/well (9HTEo- and CFBE41o-) or 1.0 × 10^5^ cells/well (C38 and IB3-1) epithelial cells in precoated 6-well tissue culture plates, 48 hours before infection. Cells were grown at 37 °C in 5% CO_2_ and, prior to bacterial infection, were incubated for 2 hours in antibiotic-free medium, containing 2% FBS. In experiments involving PDIs inhibitors, epithelial cells were pretreated with the various compounds for 30 minutes before adding bacteria and maintained thorough the infection, unless otherwise stated. Bacteria were grown to mid-exponential phase (OD_600_ of 0.3–0.4) and then diluted in antibiotic-free medium containing 2% FBS, with or without 10 mM GSH, 1 mM DTNB, 1 mM DTT, 0.3 mM bacitracin, 10 μM 16F16, 30 μM rutin, 50 μg/ml of antibody against P4HB or ERp57, or 20 and 40 μM EGCG. The bacterial suspension was used to infect semi-confluent cell monolayers at a multiplicity of infection (MOI) of 10 bacteria/cell. Bacterial pre-treatments with 1 mM DTNB or 1 mM DTT were carried out in antibiotic free medium for 1 hour. After centrifugation, pretreated bacteria were resuspended in antibiotic-free medium to infect 9HTEo- cells for 1 hour.

Invasion assays were performed by using the ceftazidime-amikacin protection assay, as described^18^. Infected monolayers were incubated for 3 hours at 37 °C in 5% CO_2_ or 1 hour in the case of DTNB. Then, infected cells were washed twice with PBS and fresh medium containing amikacin (1 mg/ml) and ceftazidime (1 mg/ml) was added to kill extracellular bacteria. After 2 hours of incubation at 37 °C, a time sufficient to ensure the killing of all planktonic bacteria^18^, monolayers were washed twice with PBS, treated with trypsin for 5 minutes at 37 °C and then lysed by the addition of 1% deoxycholic acid. Intracellular bacteria were quantified by plating serial dilutions of the lysates on PIA plates. Invasion efficiency was calculated as the number of intracellular bacteria recovered after cell incubation in presence of antibiotics divided by the number of bacteria added to each well. To evaluate the number of total bacteria associated to cells (adherent + intracellular), infected monolayers were gently washed with PBS 4 times and bacteria were isolated by lysing cells with trypsin and deoxycholic acid and quantified by plating serial dilutions of the lysates on PIA plates. Each invasion assay included at least three independent replicates.

### Immunofluorescence staining and microscopy

To detect the presence of ERp57 on the cell surface, cells were stained at 4 °C following a procedure that prevents the endocytosis of the antigen-antibody complex and minimizes lateral mobility of bound antibody along the plane of the plasma membrane.

After an incubation of 5 hours in antibiotic-free medium containing 2% FBS, 9HTEo- cells were washed four times with PBS and 0.1% sodium azide and incubated at 4 °C for 30 minutes in 10% FBS, 0.1% sodium azide to prevent non specific binding of antibodies. Cells were then incubated at 4 °C for 1 hour with a mouse monoclonal antibody against ERp57 (SC-23886, Santa Cruz) diluted 1:100 in PBS/0.1% sodium azide. After extensive washing with PBS/0.1% sodium azide, bound antibodies were detected by incubating slides with Alexa Fluor® 488 Anti-Mouse IgG (A21202, Life Technologies) diluted 1:500 for 1 hour at 4 °C. 9HTEo- cells were then fixed in 4% paraformaldehyde diluted in PBS/0.1% sodium azide and the nuclei were counterstained with Hoechst.

Total staining of ERp57 was obtained by fixing 9HTEo- monolayers in cold methanol for 10 minutes at −20 °C and permeabilizing cells with 0.1% Triton X-100. To prevent non-specific binding of antibodies, slides were incubated with 5% BSA (Biowest) in PBS for 1 hour at room temperature. Cells were then incubated with the mouse monoclonal antibody SC-23886 against ERp57 diluted 1:100 in 5% BSA overnight at 4 °C. Bound antibodies were detected by incubating slides with Alexa Fluor® 488 Anti-Mouse IgG (Life Technologies) diluted 1:500 for 1 hour at room temperature and the nuclei were counterstained with Hoechst.

Immunostained cells were mounted and visualized under a confocal microscope (Olympus Fluoview 1000) and excited at 488 nm with an Argon-ion laser and at 405 nm with a diode laser. Orthogonal projections XZ and YZ obtained from confocal system acquisitions were elaborated with software Imaris 7.1 (Bitplane).

### Detection of external plasma membrane thiols

For detection of external plasma membrane thiols, cells were seeded in precoated 12-well tissue culture plates and grown at 37 °C in 5% CO_2_. After 48 hours of incubation, monolayers were incubated for 2 hours in antibiotic-free medium containing 2% FBS. After 3 hours of incubation with or without 1 mM DTT or 1 mM GSH or after 1 hour with or without 1 mM DTNB, the monolayers were washed and incubated in PBS alone or with 10 μM Alexa fluor 488 C_5_-maleimide (Molecular Probes) for 30 minutes at 37 °C. Subsequently, the cells were washed and scraped in PBS. Labeled thiols were detected by cytofluorimetric analysis using a BD FACScalibur.

### Cytokine analysis

The amount of cytokine mRNA was determined by quantitative real-time PCR. Total RNA from cells was isolated using the High Pure RNA isolation kit (Roche, Mannheim, Germany). Reverse transcription (RT) was performed using the High Capacity cDNA Archive kit (Applied Biosystems, Foster City, CA, USA). Real-time qPCR analyses were carried out blindly by the QuantiGene Service of the Italian Cystic Fibrosis Research Foundation, using established procedures^66^. The levels of IL-8 (diluted 1:20) and IL-6 in supernatants were also measured by ELISA (R&D Systems, UK), according to the manufacturer’s instructions.

### siRNA-mediated knockdown

siRNA were purchased from Qiagen. For each gene, four different siRNA were used: SI02662100, SI02662639, SI00018501, SI03026191 for PDIA1; SI02654771 SI02654778 SI00075656 SI00075663 for ERp57. As a control, we have used AllStars Negative Control siRNA. The siRNA knockdown experiments were carried out by plating 1.5 × 10^5^ cells in six-well plates (3 wells for each gene silencing) and transfecting for 8 hours with 30 pmol siRNA using Lipofectamine RNAiMAX (Invitrogen) in Opti-MEM I-medium (Invitrogen) and MEM devoid of antibiotics. Medium was changed and the day after each well was split 1:4 in a 24-well plate and incubated for further 48 hours. Six wells were used for *Burkholderia* invasion assays and the others to verify knockdown by western blotting.

### Data analysis

Statistical analysis was performed using the SigmaPlot version IV software (Systat Software, San Jose, CA). Data describing the effect of chemical compounds (GSH, DTT, DTNB, bacitracin, 16F16, rutin and EGCG) on the ability of *B. cenocepacia* to adhere or invade epithelial cells were analyzed using the non-parametric Kruskal–Wallis test, completed with the Dunn’s Multiple Comparison post-hoc test when required. Data from invasion experiments in cells treated with siRNAs or antibodies were analyzed using one way ANOVA and completed with the Bonferroni post-hoc test. The Student’s t-test was used to analyze the statistical significance of the variation of cytokine expression between infected cells and infected cells with each PDI inhibitor. Differences in IL-6 and IL-8 release were analyzed using a two way ANOVA test and completed with the Bonferroni post-hoc test. Data are expressed as mean ± standard deviation. *p* values <0.05 were considered significant. The control values in infections experiments carried out with *B. cenocepacia* LMG 16656 and 9HTEo- cells are the average of a large number of independent experiments (55 invasion assays and 76 measures of the total number of bacteria associated to cells). The values ± SD describing the effect of each compound on bacterial ability to infect epithelial cells are the average of a number of independent experiments, each one including replicates, reported in the legends to figures.

## Additional Information

**How to cite this article**: Pacello, F. *et al.* An ERp57-mediated disulphide exchange promotes the interaction between *Burkholderia* *cenocepacia* and epithelial respiratory cells. *Sci. Rep.* **6**, 21140; doi: 10.1038/srep21140 (2016).
